# Fibroblasts: the neglected cell type in peripheral sensitisation and chronic pain? A review based on a systematic search of the literature

**DOI:** 10.1136/bmjos-2021-100235

**Published:** 2022-01-18

**Authors:** Naomi Shinotsuka, Franziska Denk

**Affiliations:** 1 Laboratory for Pharmacology, Pharmaceuticals Research Center, Asahi Kasei Pharma Corporation, Izunokuni, Shizuoka, Japan; 2 Wolfson Centre for Age-Related Diseases, Institute of Psychiatry, Psychology and Neuroscience, King's College London, London, UK

**Keywords:** fibroblasts, pain

## Abstract

Chronic pain and its underlying biological mechanisms have been studied for many decades, with a myriad of molecules, receptors and cell types known to contribute to abnormal pain sensations. Besides an obvious role for neurons, immune cells like microglia, macrophages and T cells are also important drivers of persistent pain. While neuroinflammation has therefore been widely studied in pain research, there is one cell type that appears to be rather neglected in this context: the humble fibroblast. Fibroblasts may seem unassuming but actually play a major part in regulating immune cell function and driving chronic inflammation. Here, our aim was to determine the breadth and quality of research that implicates fibroblasts in chronic pain conditions and models.

**Objectives:**

We set out to analyse the current literature on this topic—using systematic screening and data extraction methods to obtain a balanced view on what has been published.

**Methods:**

We categorised the articles we included—stratifying them according to what was investigated, the estimated quality of results and any common conclusions.

**Results:**

We found that there has been surprisingly little research in this area: 134 articles met our inclusion criteria, only a tiny minority of which directly investigated interactions between fibroblasts and peripheral neurons.

**Conclusions:**

Fibroblasts are a ubiquitous cell type and a prominent source of many proalgesic mediators in a wide variety of tissues. We think that they deserve a more central role in pain research and propose a new, testable model of how fibroblasts might drive peripheral neuron sensitisation.

**Strengths and limitations of this study**

- We took a systematic approach to what would ordinarily be a narrative review in order to obtain a more unbiased view on the state of the field.
- We defined inclusion and exclusion criteria ahead of time in our study protocol and considered randomisation, blinding and sample size calculations during our data extraction step.
- Our approach was limited by the heterogeneity of the underlying study types, making it impractical to perform meta-analytical analyses on parameters like effect size.
- Evaluating study quality against such a heterogenous backdrop is non-trivial. We attempted to mitigate this by employing a variety of quality control measures, each with their own advantages and drawbacks.

## Introduction

Pain is an important biological response that allows living organisms to escape from danger or prevent injury. In contrast, when pain becomes chronic, it stops serving its evolutionary purpose and very negatively impacts the quality of life of many patients.^1–3^

The mechanisms underlying the transition from acute to chronic pain have been extensively investigated at preclinical level in many painful diseases including neuropathies, various forms of arthritis and headache.^4–9^ Results suggest that chronic pain is a complex, multilevel phenomenon with pathological processes occurring at all levels of the nervous system, including the peripheral sensory neuron, the spinal cord and the brain.^1^ Studies have also indicated that non-neuronal cells can be critical for the induction and maintenance of chronic pain conditions.^10 11^ For instance, cytokines and chemokines released from macrophages and other immune cells during inflammation are thought to be crucially important for the establishment of peripheral sensitisation—the process by which sensory neurons become hypersensitive or spontaneously active in a pain state.^10 12 13^

One cell type, the study of which has been rather neglected in this context, is the fibroblast. Fibroblasts were originally identified by their spindle-shaped morphology as non-epithelial, non-immune cells in connective tissues.^14^ They are of mesenchymal lineage and were at first investigated in the context of their extracellular matrix (ECM)-related functions, which include collagen synthesis (both inside and outside the cell) and ECM remodelling. However, since then, it has become clear that fibroblasts are a heterogeneous population of cells, capable of engaging both tissue-specific and tissue-independent mechanisms to majorly impact the local tissue environment, as well as disease outcomes in chronic infection, inflammation and cancer.^15^ For instance, and of relevance to many painful conditions, it has been found that fibroblasts can secrete cytokines and chemokines that can regulate the response of infiltrating leucocytes.^16 17^ Moreover, just like innate immune cells, they can detect damage-associated and pathogen-associated molecular patterns,^18 19^ therefore acting as primary sentinel cells helping to protect the host.

Considering the intimate relationship between fibroblasts and the immune system, it is therefore unsurprising that, already two decades ago, they were included on a list of cells thought to be capable of inducing peripheral neuron sensitisation.^20^ Since then, however, they seem to have engendered little interest, with the exception, perhaps, of synovial fibroblasts in the knee^21^ and recent pioneering work on fibroblasts taken from patients with small fibre neuropathy and fibromyalgia.^22–24^ However, fibroblasts are a key component of our body’s inflammatory response,^25–27^ and abnormal fibroblast function has been implicated in painful immune-mediated diseases like arthritis.^28–30^ They therefore seem a sensible cell type to explore in the study of peripheral sensitisation.

In this article, we have conducted a systematic search of the literature to assess the breadth and quality of the evidence that the field has collected on this topic to date. We compiled a review protocol to help us identify any already available studies examining the role for fibroblasts in the development or maintenance of chronic painful conditions. We find that studies examining direct interactions between fibroblasts and neurons in the context of pain are surprisingly rare, especially given the prominent role of fibroblasts in chronic inflammation^31–33^ and their ability to produce known proalgesic mediators like nerve growth factor (NGF) and interleukin (IL)-6.^34–36^

## Methods

We prepared and registered a study protocol on the Open Science Framework on 6 May 2020. A second version with updates to the first introductory page was deposited on 8 October 2021. In the following, we summarise further the contents of the first version of our protocol and highlight when we deviated from it.

### Literature search

Our focus was on any original articles which mention fibroblasts in the context of pain or painful conditions. We searched PubMed using EndNote with the search strings listed in table 1. Review articles were excluded from our search and duplicates were removed—again via EndNote.

**Table 1:** Search strings

Condition	Search terms
Osteoarthritis	(“fibroblasts”(MeSH Terms] OR “fibroblasts”(All Fields] OR “fibroblast”(All Fields)) AND (“osteoarthritis”(MeSH Terms] OR “osteoarthritis”(All Fields)) AND (“pain”(MeSH Terms] OR “pain”(All Fields)) NOT review [Publication Type)
Rheumatoid arthritis	(“fibroblasts”(MeSH Terms] OR “fibroblasts”(All Fields] OR “fibroblast”(All Fields)) AND (“arthritis, rheumatoid”(MeSH Terms] OR (“arthritis”(All Fields] AND “rheumatoid”(All Fields)) OR “rheumatoid arthritis”(All Fields] OR (“rheumatoid”(All Fields] AND “arthritis”(All Fields)) AND (“pain”(MeSH Terms] OR “pain”(All Fields)) NOT review [Publication Type)
Neuropathic pain	(“fibroblasts”(MeSH Terms] OR “fibroblasts”(All Fields] OR “fibroblast”(All Fields)) AND (neuropathic [All Fields)) AND (“pain”(MeSH Terms] OR “pain”(All Fields)) NOT review [Publication Type)
Nociceptive pain	(“fibroblasts”(MeSH Terms] OR “fibroblasts”(All Fields] OR “fibroblast”(All Fields)) AND (nociceptive [All Fields)) AND (“pain”(MeSH Terms] OR “pain”(All Fields)) NOT review [Publication Type)
Inflammatory pain	(“fibroblasts”(MeSH Terms] OR “fibroblasts”(All Fields] OR “fibroblast”(All Fields)) AND (inflammatory [All Fields)) AND (“pain”(MeSH Terms] OR “pain”(All Fields)) NOT review [Publication Type)
Musculoskeletal pain	(“fibroblasts”(MeSH Terms] OR “fibroblasts”(All Fields] OR “fibroblast”(All Fields)) AND (musculoskeletal [All Fields)) AND (“pain”(MeSH Terms] OR “pain”(All Fields)) NOT review [Publication Type)
Back pain	(“fibroblasts”(MeSH Terms] OR “fibroblasts”(All Fields] OR “fibroblast”(All Fields)) AND (“back”(MeSH Terms] OR “back”(All Fields)) AND (“pain”(MeSH Terms] OR “pain”(All Fields)) NOT review [Publication Type)
Chronic pain	(“fibroblasts”(MeSH Terms] OR “fibroblasts”(All Fields] OR “fibroblast”(All Fields)) AND (chronic [All Fields)) AND (“pain”(MeSH Terms] OR “pain”(All Fields)) NOT review [Publication Type)
Fibromyalgia	(“fibroblasts”(MeSH Terms] OR “fibroblasts”(All Fields] OR “fibroblast”(All Fields)) AND (“fibromyalgia”(MeSH Terms] OR “fibromyalgia”(All Fields)) NOT review [Publication Type)
Headache	(“fibroblasts”(MeSH Terms] OR “fibroblasts”(All Fields] OR “fibroblast”(All Fields)) AND (“headache”(MeSH Terms] OR “headache”(All Fields)) NOT review [Publication Type)
Migraine	(“fibroblasts”(MeSH Terms] OR “fibroblasts”(All Fields] OR “fibroblast”(All Fields)) AND (“migraine disorders”(MeSH Terms] OR (“migraine”(All Fields] AND “disorders”(All Fields)) OR “migraine disorders”(All Fields] OR “migraine”(All Fields)) NOT review [Publication Type)

### Screening and study selection

Screening was performed independently by the two authors using the CAMARADES NC3R-funded SyRF platform (http://syrf.org.uk/).^37^ Inclusion was determined using titles and abstracts in the first instance. If a decision could not be made on these alone, the full text of the study was accessed. We did not involve a third reviewer, as originally planned in our protocol. Rather, any articles where there was disagreement between the two screeners were rescreened and, if disagreement persisted, discussed until an agreement could be reached. We included studies the primary aim of which was to investigate either pain or fibroblasts in painful or inflammatory conditions. Any type of original article that met these criteria was included, whether the work conducted was in vivo, in vitro or in silico. Reviews were excluded. We also excluded studies that mention fibroblasts or pain only in passing. If in doubt as whether these criteria were applicable, both screeners were instructed to err on the side of inclusion in this first round of selection.

We next performed a second round of screening, assessing the full text according to the same inclusion/exclusion criteria outlined previously. Deviating from our original study protocol, only one reviewer performed this screening. However, if a decision could not be reached, the article was examined by a second independent reviewer. Articles for which a full text was not available through King’s College London’s licensing agreements were excluded from the data extraction.

A deliberate choice was made to keep our inclusion criteria broad because our review was designed to capture the full breadth of scientific studies in this area.

### Data extraction

Articles which passed our two rounds of screening were included in our data mining step. From each study, we extracted a set of criteria (table 2) for all individual experiments that related to the role of fibroblasts and fibroblast–neuron interactions.

**Table 2:** Categories for data extraction

	Category	Category options
1	Type of study	In silico, in vitro, in vivo
2	Species	For example, human, rabbit, rat, mouse
3	Direct measurement of pain	Yes or no; if so, which method was undertaken? Options: pain behaviour in animals, patient’s pain assessed in clinical practice, Visual Analogue Scale, Numerical Rating Scale or sensory testing
4	Direct interaction*	Yes or no; if not, which cells or molecules were investigated? Options: neurons, fibroblasts or cytokines. How was the direct interaction measured? Options: in the same experiment, in same paper or only mentioned in the text, but not explored experimentally
5	Experimental technique	For example, histological staining, western blot, quantitative PCR, PCR, ELISA, bulk RNA-seq, FACS, electrophysiology, animal behaviour, Ca^2+^ imaging
6	Mention of blinding	Yes or no
7	Mention of randomisation	Yes or no
8	Mention of power calculations	Yes or no
9	n numbers	n number used per group
10	P values	P value for individual experiment
11	Quality score†	0–3 (0, not qualified to judge; 1, low; 2, average; 3, high)
12	Disorder	For example, rheumatoid arthritis, osteoarthritis, tendinopathy
13	Type of intervention	For example, which model was used? was there an experimental manipulation, for example, antitumour necrosis factor?
14	Note	Short summary of experimental result

*Direct interaction means that both fibroblasts and neurons/pain were assessed, detected or measured in a single experimental output, for example, if the both neurons and fibroblasts were counted in the same tissue sections. Cases in which one cell type was manipulated and the other one was evaluated were also considered to be direct interactions.

†In addition to the objective quality scores recorded as items 6 to 10, we assigned a subjective quality score based on our experience of a particular experimental technique, assessing for instance, the quality of an image, extreme variability in the data pointing towards a possible lack of power, or lack of controls.

We had initially planned to extract n numbers, p values and effect sizes. Our study protocol anticipated that in this largely preclinical literature, effect sizes would have to be estimated from the graphs provided. Not only did this turn out to be true but also we encountered several issues that meant we had to settle on the extraction of n numbers and p values only. Specifically, we ultimately deemed the process of effect size estimation to be too time consuming and inaccurate, with many articles not providing detailed enough scales or clear measures of variability in their illustrations.

To assess experimental quality, we considered whether the authors mentioned blinding, randomisation or power calculations in relation to each of the individual experiments they described. However, given the known lack of reporting in the preclinical literature,^38 39^ we also implemented two other measures, in the hope of being able to intersect different scores to gain quantifiable data on the quality of the articles we examined.

First, we used a very rough indication of journal quality by obtaining the SCImago Journal Rank (SJR) score (http://www.scimagojr.com)^40^ for all the articles we included. The SJR score measures the number of citations a journal receives within a field, taking into account prestige. It is based on Scopus data and calculated as follows: the average number of weighted citations received by a given journal in a year is divided by the number of documents published in in the previous 3 years. A citation receives more weight if it is in another prestigious journal than if it is in a less prestigious journal, with the determination of ‘journal prestige’ the result of an iterative algorithm developed by SCImago.^41^

Second, we assigned a subjective quality score to every relevant experiment, with the first author of this study assigning a score between 0 and 3 (0, screener not qualified to judge; 1, low-quality data; 2, average-quality data; 3, high-quality data). The second author spot-checked 7/133 articles (75/596 experiments) for these scores, and the agreement between scores correlated at 0.87 (Spearman’s correlation). These subjective scores were designed to mimic a trained preclinical scientist reading and judging a paper based on their own laboratory and scientific experience. As such, they are not directly replicable and are vulnerable to the same biases that individual scientists are vulnerable to when examining preclinical data. For example, an individual scientist may be overconfident or underconfident when determining whether they are qualified to judge a particular experiment.

## Results

### Article search and inclusion

**Figure 1:**
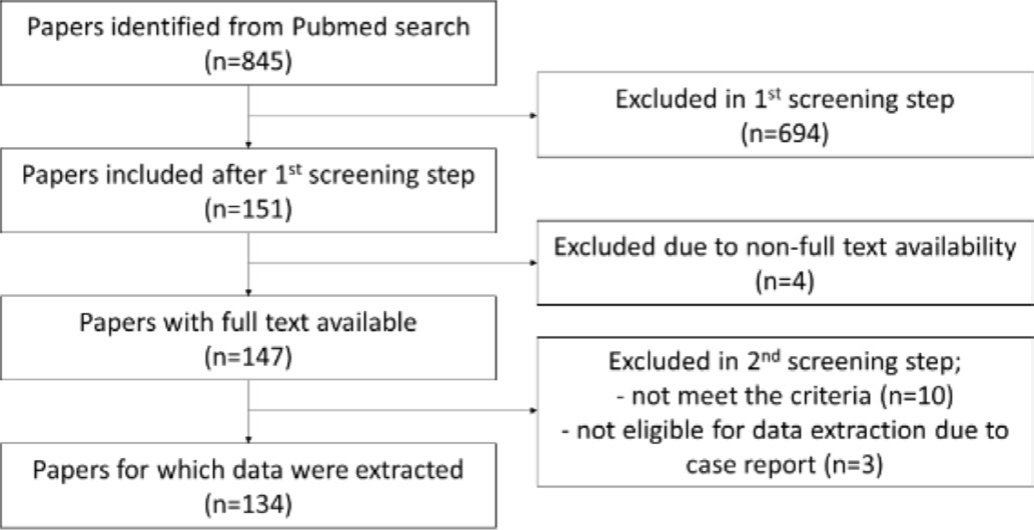
Flowchart of exclusion or inclusion of identified papers. The total number of identified papers by search strings (see table 1) from PubMed was 845. Screening results are displayed in the flowchart. At the end, 134 papers remained for data extraction.

To collect all articles which mentioned fibroblasts in the development or maintenance of chronic painful conditions, we searched PubMed on 31 May with the search strings listed in table 1. A total of 845 papers were identified, once review articles and duplicates had been excluded (figure 1). Of these, 151 publications passed our first title and abstract screen. This meant that two independent reviewers had to deem the articles to have investigated either pain or fibroblasts in painful or inflammatory conditions. Articles in which fibroblasts or pain were mentioned only in passing were excluded. After a second-stage full-text screen, 134 original articles were included for data extraction. The remaining 17 were excluded for the following reasons: unavailability of full text (4 articles), inclusion/exclusion criteria not met in the full text (10 articles) or unsuitability for data extraction due to case report format (3 articles).

We extracted and categorised the data from 134 included articles in a file deposited on the Open Science Framework, https://osf.io/m24gd/) according to the criteria in table 2. Together, they contained results from 596 individual experiments, with data derived from human (385 experiments), rats or mice (184 experiments) and various other species. In vitro and expression studies were predominant, with only 72 in vivo experiments, most of which measured animal behaviour in various disease models. The vast majority of the data was published after the year 2000, with single-digit numbers of papers appearing yearly from 2000 to 2010. Beyond 2010, the number of publications appeared to jump considerably, ranging from 11 to 29 papers every 2 years from 2012 to 2020.

In the following, we will summarise our results in more detail, reporting what scientists have already published on the link between fibroblasts and pain; we will highlight areas of agreement and identify current gaps in knowledge.

### Half of all published work on fibroblasts in the context of pain has focused on protein analysis

**Figure 2:**
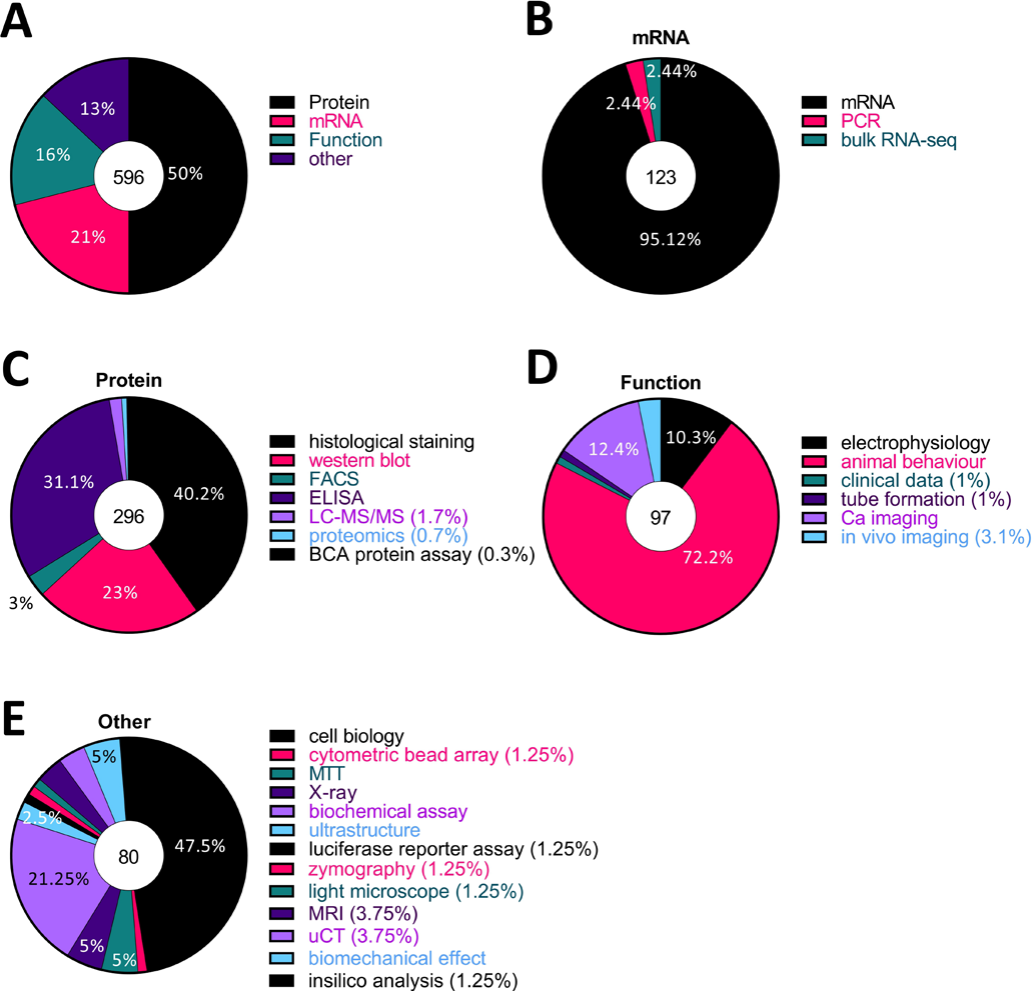
Half of all published research on fibroblasts and pain used protein analysis, mostly via histological staining, western blot and ELISA. (A) All experiments were categorised by technique and classed into four groups: ‘protein’, ‘mRNA’, ‘function’ and ‘other’, based on what was measured. (B–E) Each pie chart shows the proportion of each experimental technique in the respective group: (B) mRNA, (C) protein, (D) function, (E) other. The number in the middle circle is the total number of experiments in each category. BCA, bicinchoninic acid assay; uCT, micro computed tomography.

To know what experimental techniques have been used to investigate the relationship between fibroblasts and pain, we categorised every experiment within the articles we extracted by method, eg, histological staining, western blot and quantitative reverse transcription PCR (RT-qPCR) and classed them into four groups according to what was measured: ‘protein’, ‘mRNA’, ‘function’ and ‘other’ (figure 2A–E). ELISA, western blot, histological staining, fluorescence-activated cell sorting (FACS), proteomics and liquid chromatography with tandem mass spectrometry (LC-MS/MS) were categorised as protein. RT-qPCR, PCR and bulk-RNA seq were categorised as mRNA. Animal behaviour, Ca^2+^ imaging, in vivo imaging, electrophysiology, tube formation and clinical data were categorised function, while any remaining methods, such as biochemical and luciferase reporter assays, ultrastructural techniques and MRI were categorised as other. We found that 50% of studies measured protein levels (figure 2A), with the three major methods used being ELISA, histological staining and western blots (figure 2C). The vast majority of studies (95.12%) classified as mRNA were qRT-PCR studies, while the vast majority of functional experiments (72.2%) consisted of animal behaviour (figure 2B, D).

**Figure 3:**
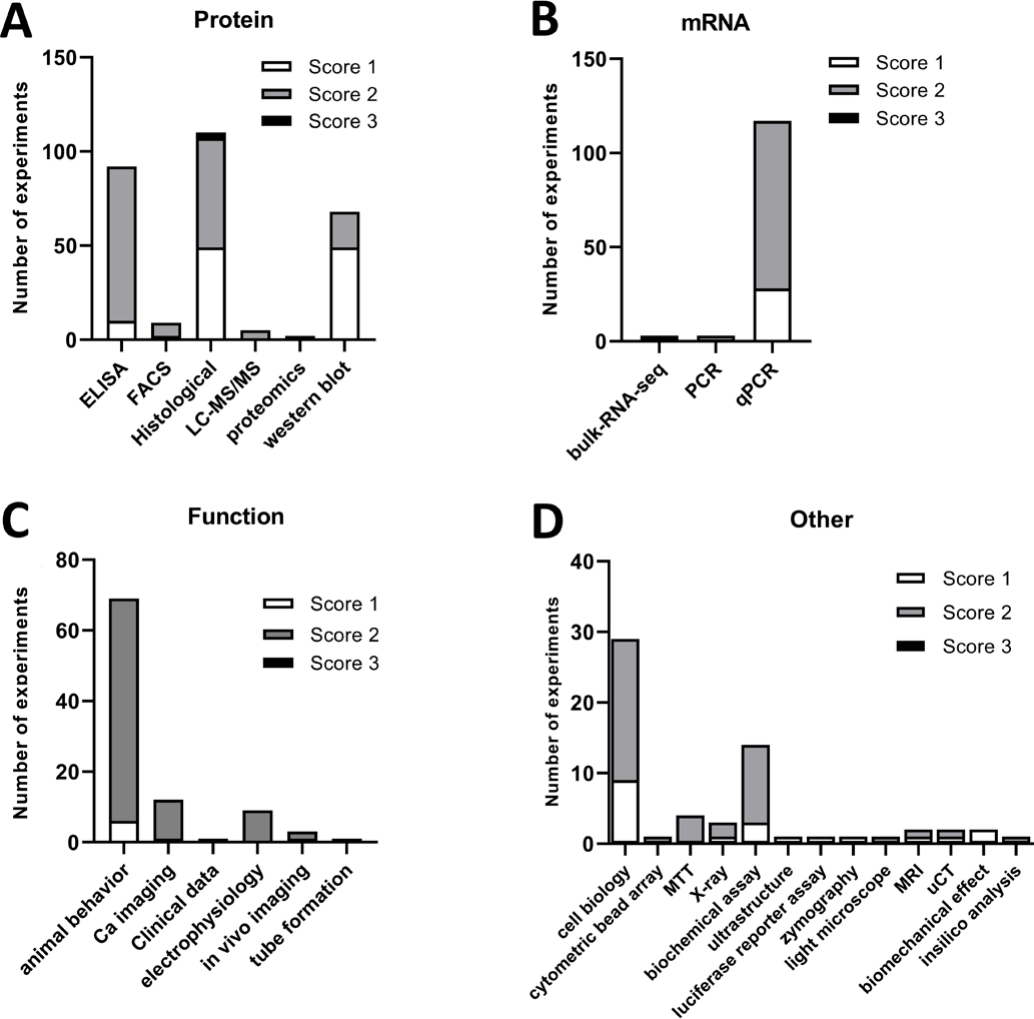
Quality score allocation to each experimental study. A subjective quality score (1, low; 2, average; 3, high) was assigned to each experiment we examined. Shown here are the number of experiments scored 1–3 across each experimental subcategory for studies examining protein (A) or mRNA (B) levels, function (C) or anything else (D).

Across these various techniques, there were some differences in the quality scores we assigned (figure 3A–D). For instance, most of the ELISA (82/92, 89.1%), quantitative PCR (qPCR) (89/117, 76.1.%) and rodent behavioural experiments (63/69, 91.3%) we examined were deemed to be of average quality (score 2). However, only half of the histological experiments (58/111, 52.3%) and a third of western blots (19/68, 27.9%) were scored to be average, while the remaining were assigned a low-quality score of 1 (50/111 and 49/68, respectively).

### Few of the experiments were deemed to be of very high technical quality, and reports of randomisation, blinding and power calculations were rare

**Figure 4:**
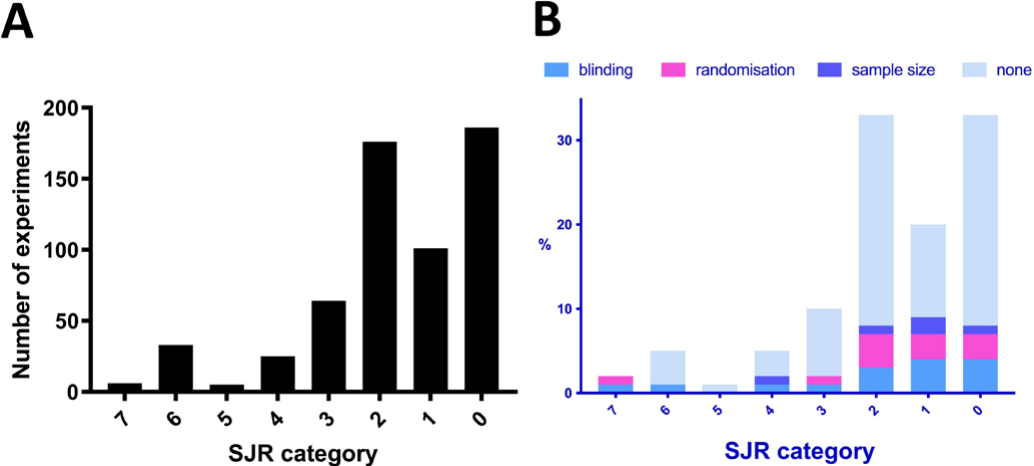
Eighty-one per cent of all studies were published in low-ranking journals according to the SJR score. (A) Experiments were grouped by SJR score: 0 (SJR score <1), 1 (1≤SJR score<1.5), 2 (1.5≤SJR score<2.5), 3 (2.5≤SJR score<3.5), 4 (3.5≤SJR score<4.5), 5 (4.5≤SJR score<5.5), 6 (5.5≤SJR score<6.5) and 7 (SJR score >6.5). The majority were published in journals with SJR score of <2.5. (B) Experiments grouped by SJR category and split according to whether they reported on blinding, randomisation, sample size calculation or none of these measures. SJR, SCImago Journal Rank.

Generally, only very few experiments (3/596, 0.5%) were assigned a quality score of 3 (‘very high quality’) (figure 3A). These all came from a single article published in *Proceedings of the National Academy of Sciences of the United States of America* (PNAS).^42^ Our scoring system was entirely subjective, however, and likely biased to pick out only very extreme ends of the spectrum. To add other proxy-measures of the quality of the articles examining fibroblasts in pain, we therefore also considered the journals they were published in (using the SJR score) and whether they mentioned features such as blinding, randomisation and sample size calculations. Mirroring our judgement to some extent, only 6 out of 596 experiments (1%) were published in a journal with an SJR score of >6.5—all within the same article in *Science Translational Medicine*^43^ (figure 4A). There were 38 experiments (6.3% of the total, across eight articles) published in journals with an SJR score above 5 (6 in *Annals of Rheumatic Diseases*, 1 in PNAS and one in *Journal of Clinical Investigation*). In contrast, 81% of all studies were found in journals with SJR scores below 2.5 (462 experiments, 109/134 articles). Moreover, only very few experiments described blinding or randomising their experimental groups (14% and 12% of 596 experiments, respectively), while even fewer (6%, 33/596 experiments described within 8 articles of the total 134) performed sample size calculations. As one might expect, blinding was most frequently discussed in the context of animal behavioural experiments (51.5% of the 33 articles which mentioned blinding and 63.0% of 27 articles assessing animal behaviour). Finally, it is important to note that failure to mention the objective measures of quality we screened for does not necessarily mean that they were not employed.

We also investigated whether there were any obvious correlations between our various quality metrics. Perhaps unsurprisingly, given the divergent nature of our measures, there were no striking correlations. For example, there seemed to be no obvious relationship between journal status and whether the authors reported on blinding, randomisation or sample size calculations (figure 4B). This is in keeping with what has been published by others^38^ and may be a consequence of both poor reporting practices in the preclinical sciences and the limitations inherent in trying to use a whole-journal metric, like SJR, in order to estimate individual study quality.

### Given the n numbers reported for the various experiments, it is likely that a lot of the literature in this field would only have been powered to detect very large effect sizes

**Figure 5:**
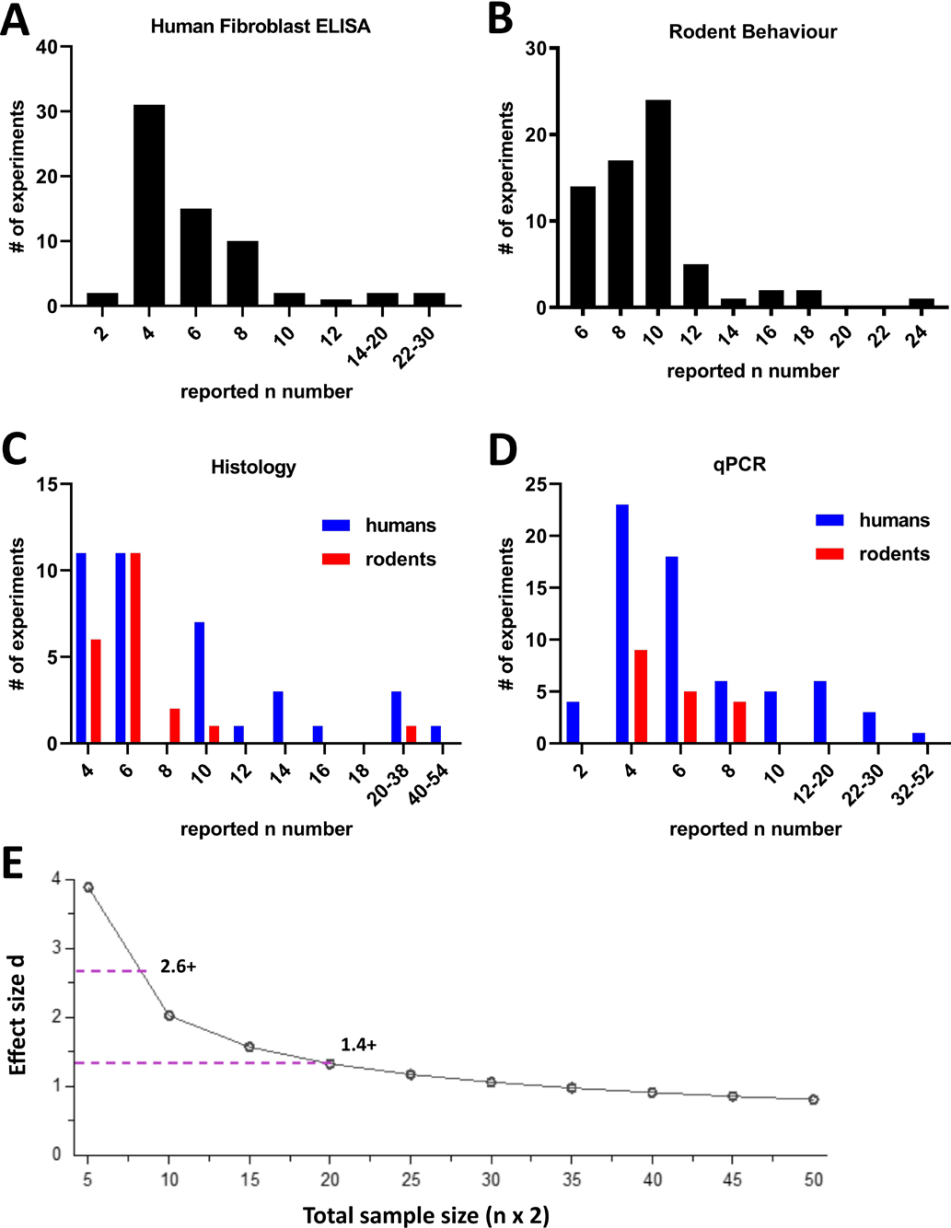
The majority of experiments were conducted with small n numbers. For the 403 experiments which reported n numbers, we plotted their distribution across the most commonly used experimental techniques: (A) human fibroblast ELISA, (B) rodent behavioural experiments, (C) histology in human or rodent tissue, and (D) qPCR with human or rodent samples. Odd numbers were counted in even number bins (eg, if n=3, it was counted in the n=4 group). Rodent groups include experiments using rats or mice. The Y axis shows the actual number of experiments in each category. (E) Sensitivity analysis for an independent samples t-test between two experimental groups with 5% alpha error probability and 80% power. Total sample size (eg, 10 for n=5) is plotted against effect size (Cohen’s d). The dotted lines indicate the minimum effect sizes one would be powered to detect under these conditions for commonly used n numbers: d=2.6 or above for n=4; d=1.4 or above for n=10. The plot was created using GPower Software. qPCR, quantitative PCR.

As part of our data extraction, we recorded the biological n used for a given experiment. First, we noted that 193 experiments did not report on the n numbers that were being used. Of those that did, we decided to particularly examine the distribution of n numbers across four of the most commonly used techniques (figure 5A–D): ELISA using human fibroblasts, rodent behaviour, qPCR (human/rodents) and histology (human/rodents).

Most ELISAs using human fibroblasts were performed with n=3–4 (31 experiments) followed n=5–6 (15 experiments) and 7–8 (10 experiments). In rodent behavioural experiments, the most commonly used n number was n=10 (21 experiments) followed by n=8 (15 experiments). While it is not possible to determine the power of these experimental studies post hoc, it is easy to appreciate that their sample sizes meant they would only have been powered to see very large effect sizes. Let us assume, for instance, that we were to conduct a simple independent samples t-test between two experimental groups, for example, comparing tumour necrosis factor (TNF) levels in fibroblasts from patients living with pain compared with those without. An n=4, that is, a total sample size of 8, would give us an 80% chance to detect effect sizes of d=2.6 and above, while a n=10, that is, a total sample size of 20, would permit us to detect effect sizes of d=1.4 and above (figure 5E). These numbers mean that to detect a difference, 95% and 83% of the patient fibroblasts, respectively, would have to show TNF levels that exceed the mean TNF levels of the control (rpsychologist.com/d3/cohend/). Indeed, using this (perhaps overly simplistic statistical scenario), only 11 experiments of all the ones recorded in figure 5C, D (2 ELISA, 1 rodent behaviour, 4 histology and 4 qPCR) would be powered to detect what is considered to be a large effect in naturalistic population scenarios, namely, Cohen’s d=0.8 or smaller (requiring n=25+).

### Most studies to date have been performed using human tissues or cells in the context of painful joints

**Figure 6:**
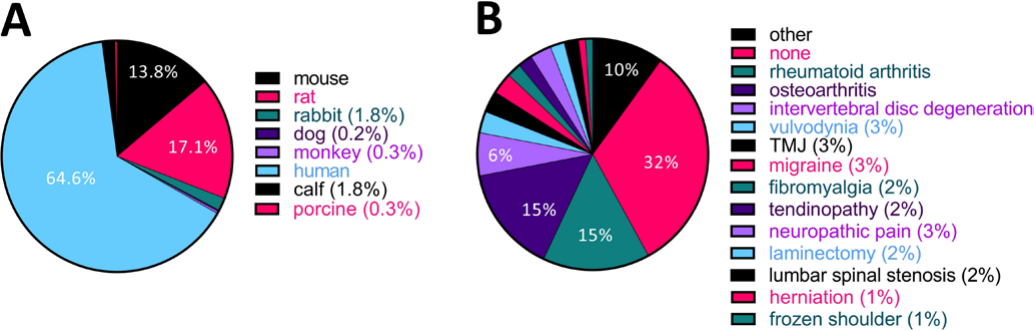
More than a third of studies to date used human samples from patients with painful joint disorders. (A) Pie chart displaying the species under investigation in the 134 articles we included in our analysis. (B) Pie chart displaying how many studies have been performed in each disease area. Animal studies were categorised according to which disease the authors of the article claimed to be modelling. If there was no mention of a specific disease in the article, it was categorised as ‘none’. The ‘other’ category includes endometriosis, meniscus tear, Fabry disease, retroperitoneal fibrosis, painful bladder syndrome, hypertrophic scarring, ankylosing spondylitis, total hip replacement, endodontic infection, hip disease, chikungunya virus disease, joint hypermobility, tooth movement, total knee arthroplasty, postoperative pain, childhood hypophosphatasia and wound healing. TMJ, temporomandibular joint disorder.

We checked what species were used for the experiments we included in our analysis. Of these experiments, 64.6% were conducted using human samples or cell lines, and only 17.1% and 13.8% were done on rats or mice, respectively (figure 6A). In many cases, human samples were collected from patients with joint disease like rheumatoid arthritis (RA) or osteoarthritis (OA). Indeed, in terms of disease areas, RA and OA were the most investigated diseases at 15% (figure 6B) out of a total of 30 conditions that were studied across the articles that met our inclusion criteria. This percentage increases to 36.8% if we consider any pain relating to joints (OA, RA, temporomandibular joint disorder (TMJ), meniscus tear, frozen shoulder, total hip replacement, hip disease, total knee arthroplasty and joint hypermobility).

### Few studies have investigated the interaction between fibroblasts and nociceptive neurons directly, and even indirectly, studies involving neurons have remained rare

We categorised each experiment into whether it measured a direct or indirect interaction between fibroblasts, neurons and/or pain perception. Studies that examined these relationships only indirectly were further subcategorised according to which cells or molecules were investigated and whether the article included separate experiments on both fibroblasts and neurons, or whether it just made reference to one of the cell types in the text.

**Figure 7:**
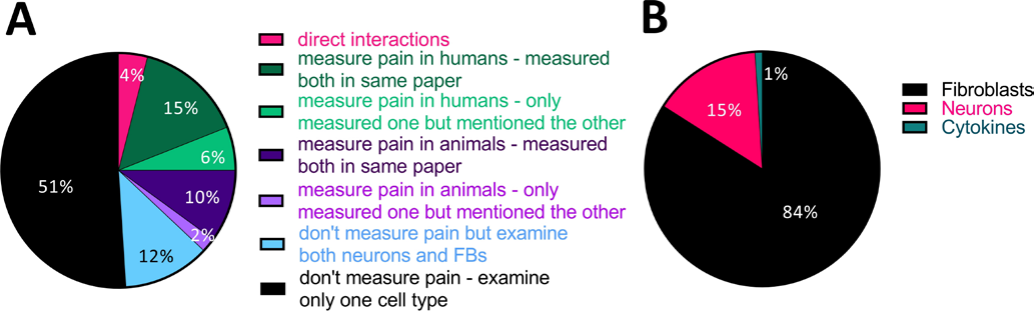
Very few studies have investigated direct interactions between fibroblasts, nerve function or pain. (A) Pie chart displaying the % of experiments categorised according to whether they examined both neurons and fibroblasts or pain and fibroblasts in the same article or not, and if so, whether any of the experiments directly connected these two cell types or fibroblasts to pain. Only 4% (24/596) did so, that is, evaluated fibroblasts and neurons together in a single experiment or manipulated the one cell type while measuring the other. (B) Of all the other experiments (572/596), the vast majority predominantly examined fibroblast function.

In support of our thesis that fibroblasts are a rather neglected cell type in pain research, a direct interaction between fibroblasts, neurons and/or pain in the same experiment was only assessed in 9/134 (ie, 6.7%) of all included articles (figure 7A). Within these nine articles, there were a total of 24 such direct experiments, spanning a variety of techniques, including measurements of neuronal activity on treatment with conditioned medium from fibroblasts, and immunostaining of fibroblast–neuron cocultures or fibroblasts in peripheral nerve. Given this diversity in experimental approaches, there are only a few common conclusions that can be drawn from the results: three articles from three independent groups^21 44 45^ reported that conditioned medium or cytosol extracts from fibroblasts in an inflammatory state caused neuronal hyperexcitability. There were also two reports of such fibroblasts causing mechanical hypersensitivity in mice, though both articles were published by the same group.^44 46^ Finally, TNF has been found to be upregulated in rat nerve fibroblasts after injury^47^ and human skin fibroblasts of individuals suffering from the pain condition fibromyalgia.^48^

In an attempt to further categorise the articles which studied fibroblasts and pain in an indirect manner, we divided their experiments into four groups (figure 7A): those that assessed pain in humans (129 experiments, 21% of the total); those that assessed pain in animals (70 experiments, 12% of the total); those that did not measure pain but looked at both cell types (72 experiments, 12% of the total); and those that did not measure pain and looked at only one cell type (301 experiments, 51% of the total, 71/134 articles).

We considered articles as having ‘assessed pain’ in humans if they included chronic pain patients on the basis of clinical diagnosis or if they directly measured pain in participants, for example, using Numerical Rating Scales. They then usually went on to isolate tissue or cell samples for the study of fibroblasts. The disorders that have been studied like this to date are varied, with papers published on 16 different diseases, including OA (seven articles), TMJ and vulvodynia (four articles each), lumbar disk degeneration (three articles) and fibromyalgia, herniation and frozen shoulder (two articles each). In two of seven OA papers,^49 50^ one of two fibromyalgia papers^22^ and all the intervertebral disc defect and vulvodynia papers,^51–57^ it was reported that fibroblasts showed increased cytokine expression. Articles on OA also reported on the over-representation of neuropeptides in patient fibroblasts, specifically calcitonin gene-related peptide (CGRP) and NGF.^49 58–60^ The latter was reported to be upregulated in synovial fibroblasts by three independent groups.^49 59 60^

Of the studies measuring nociception in animals, there were an equivalent number of articles modelling arthritis (OA (six articles) and RA (seven articles)) and injecting proinflammatory substances or mediators into skin (six articles). There were also three and four articles, respectively, on neuropathic pain and migraine. Most of this work was focused on a particular target and described its proanalgesic/or antianalgesic properties.

Finally, the vast majority of articles examining indirect interactions were focused on experiments involving fibroblasts, with only 15% of them focused on neurons (figure 7B).

### To date, there is agreement about fibroblasts modulating the expression of prominent proalgesic mediators in response to stimulation

As discussed in the Introduction section, fibroblasts release cytokines and chemokines which could impact neurons both directly and indirectly via immune cell types. To reveal how many studies are investigating fibroblasts in the context of secreting immune-modulatory substances, we therefore identified all articles which reported a modulation in the release of four critical neuronal mediators: TNF, NGF, IL-6 and CGRP (table 3). We also noted which neuropeptides, cytokines or receptors were reported to be responsible for the production of these molecules.

**Table 3:** Many experiments and articles reported a modulation in the release of critical neuronal mediators in response to a large variety of interventions

Experiments (n)	Articles (n)	Inducer (+)/suppressor (–)	Molecule linked to modulation
TNF			
34	19	+IL-1a, IL-1b [2], LPS [2], nerve injury or inflammation (CCI, DMM, OA, microinjury at ligament flavum, monosodium urate), human disorder (FM, RA, intervertebral disc degeneration, degenerative lumbar spondylolisthesis, TMJ meniscus tears), infection (*Candida albicans*)	+PKCgamma (KO), Wnt (inhibitor), macrophage (pharmaceutical depletion), Cyr61 (shRNA)
		−TGF-b1	−Foxo3 (siRNA), AMPK (inhibitor), p38 (inhibitor, siRNA), miR-92a (mimic), herbal remedy (*Aralia continentalis* Kitag., *Betula platyphylla*, Huzhang Tongfeng granule), platelet-rich plasma, vitamin E
NGF			
19	7	+IL-1b [3], TNF [2], injury (DMM [2], OA, cartilage injury, muscle injury), human disorder (OA [3])	+FGF2 (KO), FGFR (inhibitor), TAK1 (inhibitor), SRC (inhibitor)
		–	− PKCgamma (KO), Cox2 (inhibitor), PGE2 (agonist), EP (agonist)
IL-6			
64	32	+IL-1a [3], IL-1b [12], TNF [2], HMGB1, bradykinin, PGE2, EP2, EP4, TLR7, norepinephrine,* EDPs, LPS [4], poly(I:C), infection (*C. albicans* [2], *C. glabrata*, *C. tropicalis*, CHIKV, HIV), zymosan, nerve inflammation (monosodium urate), human disorder (vulvodynia [3], RA [3], OA, total knee arthroplasty, ligament injury, FM, frozen shoulder)	+IKKkb (OE, KO), NFkb (inhibitor) [2], Dectin1 (decoy ligand, siRNA), Wnt (inhibitor), bradykinin receptor (isRNA, inhibitor), macrophage (pharmaceutical depletion), Cox2/Cox (inhibitor) [2], IL-1R (antagonist), PKA (inhibitor)
		−Dexamethasone, cannabinoid 2, phosphatidylserine, dihydroartemisinin derivative, benzylideneacetophenone derivative, gabapentin	−Cannabinoid R2 (agonist), glucocolticoid receptor (siRNA), herbal remedy (piperine, *Aralia continentalis* Kitag., WIN-34B, *Betula platyphylla*, Huzhang Tongfeng granule), platelet-rich plasma, Cyr61 (shRNA)
CGRP			
5	2	+PGE2, muscle injury, human disorder (OA)	–
		–	–

*Reported in headache. The numbers in square brackets indicate if a factor was used in more than one article.

CCI, chronic constriction injury; CHIKV, chikungunya virus; DMM, destabilisation of the medial meniscus; EDP, elastin-derived peptide; FM, fibromyalgia; KO, knock out; OA, osteoarthritis; OE, overexpression; PGE2, prostaglandin E2; RA, rheumatoid arthritis; TMJ, temporomandibular joint disorder; TNF, tumour necrosis factor.

## Discussion

Stromal cell immunology has become a very prominent field over the past decade but has yet to make a significant impact on pain research. Here, we took a systematic approach to determine what is already known about how fibroblast (dys-)function is connected to peripheral neuron hypersensitivity and chronic pain. Our methods were designed to provide a non-prejudicial overview of the literature in this area and confirmed what a superficial reader might suspect: collectively, we know very little about fibroblasts and their role in pain.

We found that the vast majority of studies in this area split into two categories: those with a more immunological bent which studied cytokines and other mediators released from fibroblasts during inflammation, and those emerging from the neuroscience literature, which tended to prioritise animal behaviour—still considered a gold standard method for evaluating nociception. Only very few articles tried to link these two elements to study the interaction between nerves, fibroblasts and pain more directly. There was great variation in the painful disorders that were being investigated, though ~30% were focused on OA and RA. Technically, most studies appeared of average quality, though the majority would likely not have been powered to see anything but very large effect sizes.

Our approach had some clear limitations. We only used a single database in our search and excluded four articles that were not available through our university subscription service. This means that we are missing some data available in this area. Moreover, while abstract screening was performed in parallel by both authors, data extraction was conducted by only one of us, making it somewhat more prone to error and bias. Finally, and probably most importantly, we were limited by the unstandardised reporting that is common in much of the preclinical literature. Consequently, extracting data like effect sizes was prohibitively complex, given our time and resource constraints. Moreover, it is difficult to interpret non-reporting in the context of study quality; for example, did authors who failed to mention blinding fail to implement this crucial experimental design aspect, or did they simply not think to mention it in their write-up? Indeed, all indicators we devised to assess study quality had significant uncertainty attached to them: objective measures, like blinding and randomisation, were under-reported; journal citation scores, like SJR, are thought to be only loosely related to individual study quality, if at all^38^; and our subjective quality score is just that: subjective and therefore not replicable.

In fact, the subjective score—as we tried to introduce it here—is not a metric we would recommend for future use. The scale was too biased towards identifying only extreme outliers, making it of too little use to offset its obvious drawback of subjectivity. For example, we found that the experiments we scored to be of particularly low quality tended to be those examining protein expression via histology or Western blot. On the one hand, this is an important finding in an area that is so far predominantly reporting on what fibroblasts do or do not express in painful conditions or disease models. On the other hand, it is also a result that is of high risk of bias: it is much easier to detect flaws in experimental techniques when provided with an actual image of the result (eg, via a western blot). Many other types of results, like rodent behavioural data, are much harder to assess, with poor reporting practices and lack of raw data essentially forcing readers to take them on faith.

When we set uncertainty about study quality aside and examine the data we collected as a whole, it is clear that they mirror what we already know from the immunology field.^25–27^ For example, sequencing results published by Zhang *et al*^35^ and Wei *et al*^61^ suggest that synovial fibroblasts upregulate a host of inflammatory mediators in RA and OA—some of which, like IL-6 and NGF, we know to be proalgesic. Nevertheless, the details of this process and how exactly it affects nociception and peripheral hypersensitivity over time remain grossly understudied. For instance, it is yet to be demonstrated whether human synovial fibroblasts from patients with RA release NGF—and whether they continue to do so in the many individuals who continue to experience pain in the absence of synovitis.^62^ We also know nothing about whether known fibroblast subpopulations in joint,^28^ skin^63^ or other tissues^64^ differentially affect nociceptor sensitisation. Finally, we lack information on how fibroblasts contribute to the immune cell dysfunction frequently demonstrated and characterised in chronic pain states.^10^

These gaps are very significant. Consider for a moment that fibroblasts are a ubiquitous cell type and that transcriptional databases would suggest that they are likely the most prominent, if not the only source of NGF and IL-6 in a wide variety of tissues. How could we not consider them more closely in the context of peripheral sensitisation? Epigenetic alterations in fibroblasts have been shown to result in their persistent dysfunction^32^—a dysfunction which could explain why nociceptors remain overactive in tissues that lack obvious signs of inflammation.

**Figure 8:**
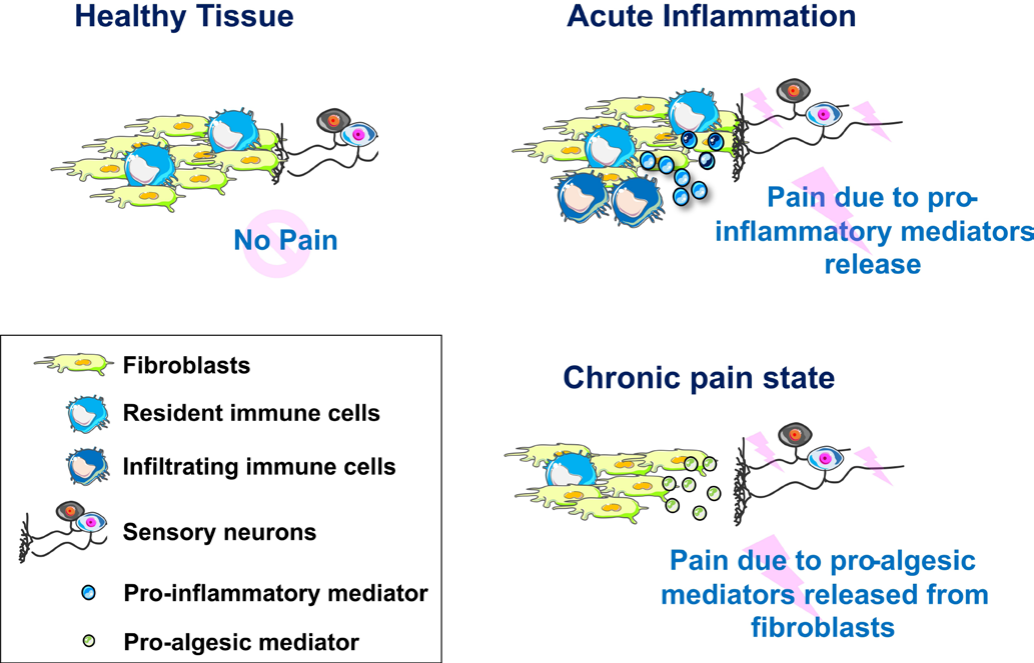
Model of fibroblast contribution to peripheral sensitisation. In healthy tissues, sensory neurons, resident immune cells and fibroblasts act together to ensure host defence. In acute inflammatory states, proinflammatory mediators released from resident and infiltrating immune cell populations will affect neuronal function directly, as well as indirectly via activation of fibroblasts (eg, tumour necrosis factor priming fibroblasts to release interleukin-6). In chronic pain states, fibroblasts might be the primary drivers of peripheral sensitisation, releasing proalgesic mediators as a result of long-term shifts in function or low-grade activation through resident immune cells.

We propose that we should include fibroblasts in our model of how nociceptor hyperactivity arises and persist over time (figure 8). Their addition allows for a range of testable hypotheses, including that fibroblast-specific knockout of NGF would be analgesic. We hope that other scientists in the pain field are intrigued by our suggestion and join us in researching this cell type—to further our understanding of peripheral pain mechanisms and ultimately benefit the many individuals living with chronic pain.

## Data Availability

Data are available in a public, open access repository. Our study protocol is available on the Open Science Framework (https://osf.io/m24gd/?view_only=0f245d2097b743048a92ad22669747ea).
